# Effect of Soil-Applied Metabolic Modulators on the Accumulation of Specialized Metabolites in *Chelidonium majus* L.

**DOI:** 10.3390/molecules30132782

**Published:** 2025-06-27

**Authors:** Maria Stasińska-Jakubas, Sławomir Dresler, Maciej Strzemski, Magdalena Wójciak, Katarzyna Rubinowska, Barbara Hawrylak-Nowak

**Affiliations:** 1 Department of Botany and Plant Physiology, Faculty of Environmental Biology, University of Life Sciences in Lublin, Akademicka 15, 20-950 Lublin, Poland; maria.jakubas@up.lublin.pl (M.S.-J.); katarzyna.rubinowska@up.lublin.pl (K.R.); 2 Department of Analytical Chemistry, Medical University of Lublin, Chodźki 4a, 20-093 Lublin, Poland; slawomir.dresler@umlub.pl (S.D.); maciej.strzemski@umlub.pl (M.S.); magdalena.wojciak@umlub.pl (M.W.); 3 Department of Plant Physiology and Biophysics, Institute of Biological Science, Maria Curie-Skłodowska University, 20-033 Lublin, Poland

**Keywords:** elicitation, secondary metabolites, isoquinoline alkaloids, greater celandine, phytochemistry

## Abstract

Various metabolic modulators have been widely used in recent years to increase the accumulation of desired secondary metabolites in medicinal plants, although most studies to date have focused on in vitro systems. Although simpler and cheaper, their potential application in vivo is still limited. Therefore, the aim of this study was to compare the effect of three chemically different elicitors (150 mg/L chitosan lactate—ChL; 10 mg/L selenium as selenite—Se; 100 mg/L salicylic acid—SA) applied to the soil substrate on some aspects of the secondary metabolism and physiological responses of *Chelidonium majus* L. Using HPLC-DAD, six isoquinoline alkaloids were identified and quantified in shoot extracts. LC-ESI-TOF-MS analysis confirmed the molecular identity of all target alkaloids, supporting the identification. The strongest stimulatory effect on the accumulation of protopine, berberine, and allocryptopine was observed with the Se and SA treatment, whereas ChL was less effective. In turn, the dominant alkaloids (coptisine and chelidonine) remained unaffected. There was also an increase in total phenolic compounds, but not in soluble flavonols. The elicitor treatments caused an increase in the antioxidant activity of the plant extracts obtained. Regardless of the metabolic modulator type, the strongest effect was generally observed on days 7 and 10 after application. No visual signs of toxicity and no effect on shoot biomass were found, although some elicitor-induced changes in the oxidative status (increased H_2_O_2_ accumulation and enhanced lipid peroxidation) and free proline levels in leaves were observed. We suggest that Se or SA can be applied to *C*. *majus* grown in a controlled pot culture to obtain high-quality raw material and extracts with increased contents of valuable specialized metabolites and enhanced antioxidant capacity.

## 1. Introduction

Medicinal plants have been playing a key role in human life since ancient times. Initially, they were used to improve general well-being, but they are currently applied in medicine and modern pharmacotherapy both directly as medicinal agents and indirectly as basic raw materials for the production of medications [1,2]. The therapeutic value and suitability of medicinal plants are mainly associated with their contents of secondary metabolites, which are characterized by a wide spectrum of pharmacological activities, e.g., from antioxidant and antimicrobial to anti-inflammatory and anticancer effects. Moreover, as a rich source of such pro-health substances as phenols, flavonoids, alkaloids, glycosides, and aromatic compounds, they are widely used not only in the pharmaceutical industry but also in the manufacture of cosmetic and food. Therefore, the content of secondary metabolites in plant raw materials is one of the most important indicators in the assessment of their quality [2,3,4,5].

In addition to their extremely useful biological activity for humans, secondary metabolites are an important element of complex plant responses to the impact of abiotic and biotic environmental factors and are most often synthesized by plants to serve defensive, signaling, or adaptive functions. This leads to changes in the overall phytochemical profile of plants and properties of plant raw material [6,7,8]. The growing number of literature reports on the impact of environmental factors on plant metabolism indicates the validity of the use of moderate and controlled stimuli in the elicitation process, which is one of the methods for optimization of the production of phytochemicals [5,7,9]. This strategy is considered one of the most effective approaches to the manipulation of plant metabolic pathways aimed to increase the efficiency of the production of desired secondary metabolites. Although elicitation is more frequently studied and used in in vitro plant cultivation, the in vivo application of this method may be simpler and more economical, especially in controlled conditions where there is no impact of interfering geographical or climatic factors. The in vivo elicitation most often entails the foliar or soil application of an elicitor solution or the conditioning of seeds [10].

The greater celandine (*Chelidonium majus* L.) from the family Papaveraceae is a perennial plant with a branched habit and a height up to 100 cm. This synanthropic species occurs in all regions of Europe and Asia. Its characteristic hollow stem bears light green pinnate leaves with lobed or serrated margins. The petiolate basal leaves with large blades are gathered in a rosette; the stem has small sessile leaves. *C. majus* blooms from April to October; it produces actinomorphic, umbellate, yellow inflorescences. Its fruit is an elongated capsule with shiny, ovoid, dark brownish-black seeds equipped with elaiosomes. All organs in this species contain large amounts of yellow–orange milky sap (latex), which is commonly used in folk medicine to treat warts, corns, eczema, fungal infections, and other skin diseases [11,12,13,14].

Unlike many medicinal plant species where phenolic compounds or terpenoids dominate the phytochemical profile, *C*. *majus* is distinguished by its specific and highly concentrated isoquinoline alkaloid content. The predominance of alkaloids not only defines the pharmacognostic identity of the species but also reflects their ecological role in plant defense mechanisms against stress factors [12,15]. Their pharmacological and ecological importance justifies a focused approach to enhance their accumulation through elicitation strategies. The pharmacopoeial raw material provided by the plants is the flowering leafy tops of celandine shoots (*Chelidonii herba*) containing no less than 0.6% of total alkaloids expressed as chelidonine equivalents. Another raw material used in medicine is the root (*Chelidonii radix*), which contains up to 3% of alkaloids. Over 30 compounds have been identified in the group of celandine alkaloids, with chelidonine, chelerythrine, sanguinarine, berberine, and coptisine as the dominant compounds. In addition to isoquinoline alkaloids, the roots of the plant contain saponins, flavonoids, tannins, and organic acids. In turn, celandine herb contains mainly 0.2–0.6% of alkaloids and flavonoids. Due to their antispasmodic, antiulcer, and choleretic effects, celandine raw materials and their extracts are most often used as ingredients in preparations for treatment of gastrointestinal and biliary tract diseases. These raw materials also exhibit anti-inflammatory, antibacterial, antifungal, antiviral, immunomodulatory, and analgesic activity. A slight sedative effect and amelioration of anaphylactic shock have been reported as well [11,12,13,15]. With their content of isoquinoline alkaloids, *C*. *majus* extracts have anticancer potential, as they have been reported to exert strong antioxidant, antiproliferative, proapoptotic, and cytotoxic effects on cancer cell lines [16,17].

In this study, we verified a hypothesis that the induction of a physiological response in *C*. *majus* plants with chemical elicitors results in changes in plant secondary metabolism and the redox balance. The effectiveness of chemically diverse compounds in the elicitation of selected specialized metabolites in controlled pot cultivation conditions was analyzed. The effects of the soil application of aqueous solutions of chitosan lactate (ChL), sodium selenite (Se), and salicylic acid (SA) were compared depending on the exposure time (4, 7, and 10 days). This study presents a novel soil-based elicitation protocol targeting isoquinoline alkaloids and phenolic compounds in greater celandine under controlled in vivo conditions. To our knowledge, this specific approach has not been previously reported and addresses the limitations of foliar application in this species due to leaf hydrophobicity. This method may contribute to a controlled increase in the level of pro-health bioactive substances, modifying the effects of celandine raw materials.

## 2. Results

### 2.1. Selected Secondary Metabolites in C. majus Shoots Grown Under Exposure to Different Elicitors

**Figure 1:**
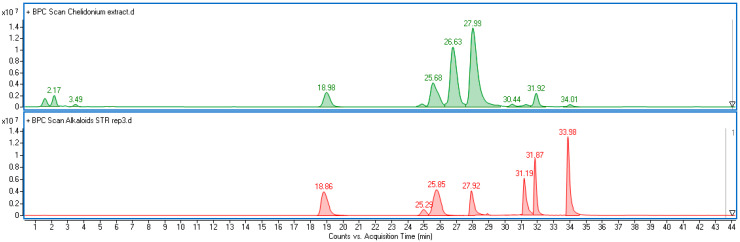
Base peak chromatogram obtained in positive ionization mode via UHPLC-ESI-TOF-MS. Green trace: extract from *C*. *majus* shoots. Red trace: mixture of standards, including protopine (R_T_ = 18.86 min), allocryptopine (R_T_ = 25.29 min), chelidonine (R_T_ = 25.85 min), coptisine (R_T_ = 27.92 min), sanguinarine (R_T_ = 31.19 min), berberine (R_T_ = 31.87 min), and chelerythrine (R_T_ = 33.98 min). Peak identities were confirmed by matching both *m*/*z* values and retention times (Table S2).

**Figure 2:**
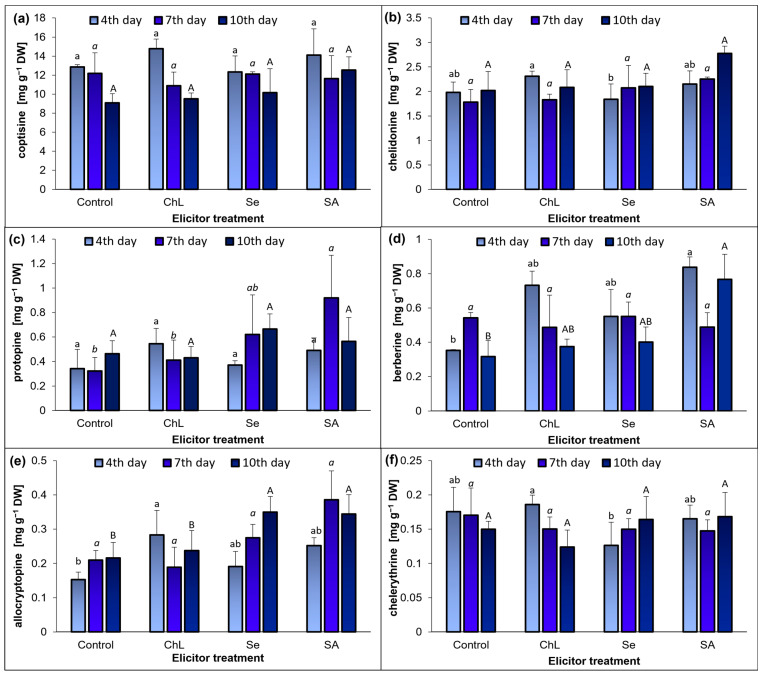
Effect of soil application of ChL—chitosan lactate, Se—sodium selenite, and SA—salicylic acid on the concentration of selected alkaloids—coptisine (**a**), chelidonine (**b**), protopine (**c**) berberine (**d**), allocryptopine (**e**), and chelerythrine (**f**)—in *C*. *majus* shoots on the 4th, 7th, and 10th day of elicitation. Control plants were treated with distilled water. Values are the means ± standard deviation, shown as positive error bars (n = 3). Different letters (lowercase, italic, or capital) indicate statistically significant differences between treatments on the same sampling day (*p* < 0.05, LSD-Fisher test). One-way ANOVA was performed independently for each time point.

Alkaloids are the most abundant specialized metabolites in the herb of *C*. *majus*, confirming their dominant role in the phytochemical profile of this species. Six isoquinoline alkaloids were identified and quantified in the extracts from the *C. majus* herb, i.e., coptisine, chelidonine, protopine, berberine, allocryptopine, and chelerythrine (Figure 1, Figures S1 and S2). The contents of these components in *C*. *majus* shoots depended on the elicitor type and the duration of the experiment (Figure 2a–f).

Coptisine was the dominant alkaloid (Figure 2a), with its content in the extracts from the control plants ranging from 9.08 to 12.86 mg g^−1^ DW on the experiment days. However, the application of the elicitors to the soil did not induce significant changes in the concentration of this compound. The next most abundant compound in the analyzed extracts was chelidonine, for which the concentration changed only slightly after the treatment with the elicitors (Figure 2b). An exception was SA, which produced a 38% increase in the chelidonine content in the analyzed extracts on day 10 after the application, compared to the control plants. In contrast, the other elicitors were not effective in stimulating the biosynthesis of this compound. The other alkaloids were present in the analyzed extracts in substantially lower concentrations (<1 mg g^−1^ DW), but their contents fluctuated to a greater extent upon exposure to the elicitors applied (Figure 2c–f). The accumulation of protopine in the aboveground parts of *C. majus* was most effectively stimulated by SA on day 7 of the experiment; in comparison with the corresponding control, the content of this alkaloid increased almost 3-fold (Figure 2c).

Concurrently, the Se solution exhibited slightly lower elicitation activity, as its use resulted in a 2-fold increase in the protopine concentration compared to the control plants. In the case of berberine, significant changes were found on days 4 and 7 after the application of the elicitors (Figure 2d). Again, the highest elicitation activity was exhibited by SA, as the content of berberine increased over 2-fold on day 4 after the application of this elicitor. The other elicitors stimulated the accumulation of this alkaloid as well, although to a lesser extent. In comparison with the control, the concentration of berberine in the analyzed extracts increased by 108% and 57% in the ChL and Se treatments, respectively. The soil application of the elicitors also resulted in significant changes in the content of allocryptopine in the *C. majus* herb extracts (Figure 2e). The highest concentration of this alkaloid (0.39 mg g^−1^ DW) and its greatest stimulation relative to the corresponding control were observed on day 7 after the application of SA (an 85% increase). The use of ChL stimulated the allocryptopine content in the analyzed extracts only on day 4 of the experiment, which increased by 86%, compared to the control. On day 10 after the application of Se and SA, the content of allocryptopine increased by 59% and 55%, respectively, in comparison with the control. The elicitors had no significant effect on the accumulation of chelerythrine, for which the level was the lowest among the quantified alkaloids (Figure 2f).

**Figure 3:**
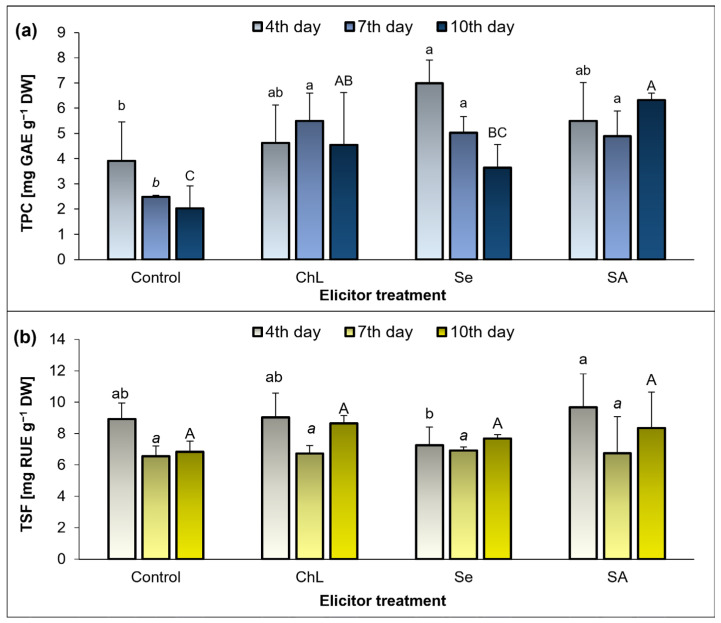
Effect of soil application of ChL—chitosan lactate, Se—sodium selenite, and SA—salicylic acid on the concentration of TPC—total phenolic compounds (**a**) and TSF—total soluble flavonols (**b**) in *C. majus* shoots on the 4th, 7th, and 10th day of the elicitor exposure. Control plants were treated with distilled water. Values are the means ± standard deviation, shown as positive error bars (n = 3). Different letters (lowercase, italic, or capital) indicate statistically significant differences between treatments on the same sampling day (*p* < 0.05, LSD-Fisher test). One-way ANOVA was performed independently for each time point. GAE—gallic acid equivalents, RUE—rutin equivalents.

The concentration of total phenolic compounds (TPCs) in the analyzed extracts from the *C*. *majus* shoots changed depending on the time after the application of the elicitors and the type of the substance used (Figure 3a). The highest accumulation of these compounds was recorded on day 4 of the experiment for the Se application variant. The TPC concentration in this treatment was 6.99 mg GAE g^−1^, which was 79% higher than in the corresponding control plants. In turn, on day 7 of the experiment, all the elicitors exhibited a similar level of efficiency, as they caused an approximate 2-fold increase in the TPC, compared to the control. The highest elicitation activity was observed on day 10 after the application of SA, which produced an over 3-fold increase in the TPC content compared to the control. The ChL solution also stimulated TPC accumulation on day 10, and the concentration of these compounds was 124% higher than in the control plants.

The total soluble flavonol (TSF) content in the analyzed plant extracts was similar in each experimental series, and the soil application of the elicitors did not exert a significant effect on the accumulation of these compounds (Figure 3b).

### 2.2. Oxidative Status of C. majus Plants Treated with Different Elicitors

Selected parameters of the plant oxidative status were determined on days 2 and 10 to assess the early and late responses of *C. majus* plants to the applied elicitors.

**Figure 4:**
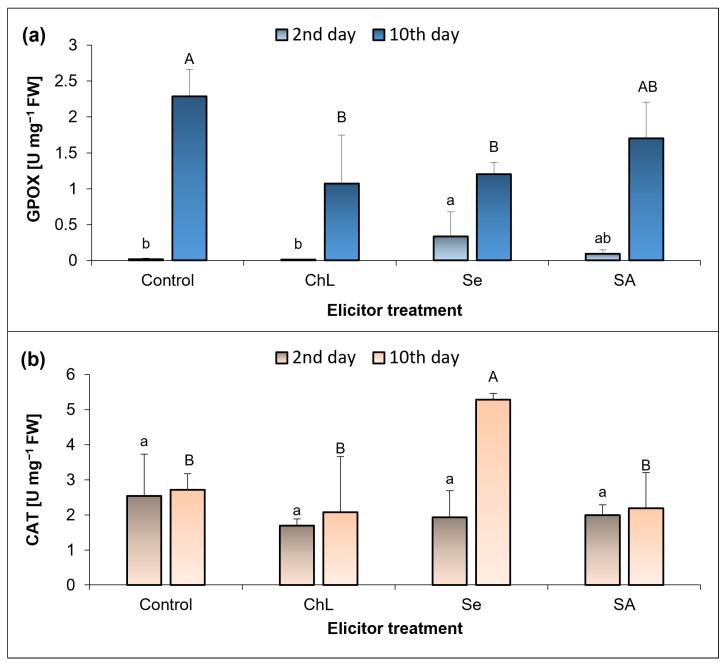
Effect of soil application of ChL—chitosan lactate, Se—sodium selenite, and SA—salicylic acid on the activity of catalase—CAT (**a**) and guaiacol peroxidase—GPOX (**b**) in *C. majus* leaves on the 2nd and 10th day of elicitation. Control plants were treated with distilled water. Values are the means ± standard deviation, shown as positive error bars (n = 4). Different letters (lowercase or capital) indicate statistically significant differences between treatments on the same sampling day (*p* < 0.05, LSD-Fisher test). One-way ANOVA was performed independently for each time point.

The activity of the selected antioxidant enzymes (CAT, GPOX, APOX) in the *C*. *majus* leaves was determined shortly after the exposure of the plants to the elicitors (day 2) and on the day of termination of the experiment (day 10) (Figure 4a,b). The GPOX activity determined on day 2 of the experiment was very low (0.02–0.33 U mg^−1^) but increased 15-fold with the soil Se treatment, compared to the control plants (Figure 4a). In turn, the activity of this enzyme on the experiment termination day increased significantly (1.07–2.29 U mg^−1^). In this case, however, the use of the elicitors caused a significant decrease in the GPOX activity, especially for the ChL and Se treatment variants (reduction by half). In general, the CAT activity (Figure 4b) was similar on both days in all the experimental series. A significant increase in the activity of this enzyme was noted only on day 10 after the application of Se (2-fold higher than in the corresponding control). No APOX activity was detected in the analyzed plant material.

**Figure 5:**
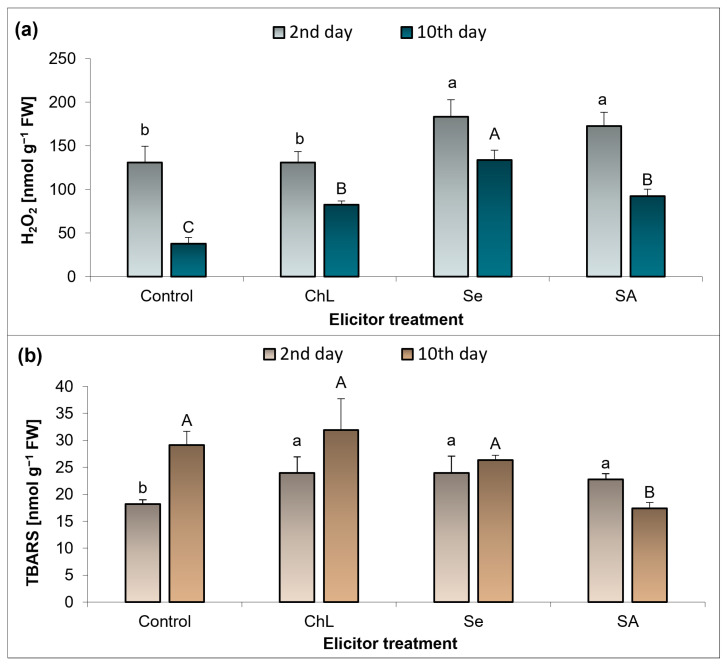
Effect of soil application of ChL—chitosan lactate, Se—sodium selenite, and SA—salicylic acid on the concentration of H_2_O_2_ (**a**) and of TBARS (**b**) in *C*. *majus* leaves on the 2nd and 10th day of the elicitor exposure. Control plants were treated with distilled water. Values are the means ± standard deviation, shown as positive error bars (n = 4). Different letters (lowercase or capital) indicate statistically significant differences between treatments on the same sampling day (*p* < 0.05, LSD-Fisher test). One-way ANOVA was performed independently for each time point.

The level of H_2_O_2_ accumulation in the *C. majus* leaves was determined shortly after the application of the elicitors (day 2) and on the day of termination of the experiment (day 10) (Figure 5a). The H_2_O_2_ level in the treatments varied depending on the duration of the plant exposure to the action of the elicitors. Initially, a 40% and 32% increase in the H_2_O_2_ concentration was noted for the Se and SA treatments, respectively, compared to the control plants. After 10 days, each of the three elicitors significantly increased the H_2_O_2_ accumulation, i.e., its concentration increased over 2-fold with the ChL and SA variants and 2.5-fold for the Se treatment, compared to the control.

The analyzed elicitors had a similar effect on the level of thiobarbituric-acid-reactive substances (TBARS) immediately after their application. On elicitation day 2, the concentration of TBARS increased by approximately 31% with the ChL and Se treatments and by 24% with the SA variant, which was statistically significant compared to the control (Figure 5b). However, on day 10 after the application of the elicitors, the SA treatment resulted in a 40% decrease in TBARS, while the other compounds had no significant effect on the level of lipid peroxidation in the *C. majus* leaf tissues.

**Figure 6:**
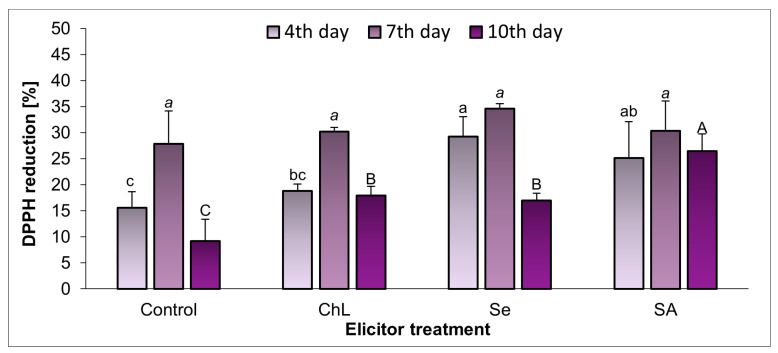
Effect of soil application of ChL—chitosan lactate, Se—sodium selenite, and SA—salicylic acid on the free radical scavenging activity of *C*. *majus* shoot extracts on the 4th, 7th, and 10th day of elicitation. Control plants were treated with distilled water. Values are the means ± standard deviation, shown as positive error bars (n = 3). Different letters (lowercase, italic, or capital) indicate statistically significant differences between treatments on the same sampling day (*p* < 0.05, LSD-Fisher test). One-way ANOVA was performed independently for each time point.

The analyzed methanol extracts of the *C. majus* herb exhibited a relatively low ability to reduce the DPPH radical, which varied depending on the elicitor used and the duration of the experiment (Figure 6). The highest antioxidant activity was observed on day 7 of the experiment (approx. 27–35%); at that time, its similar levels were determined regardless of the experimental series. At the other experiment time points, the antioxidant activity increased under the influence of the elicitors. The greatest changes relative to the control plants were noted on day 10 after the application of SA (an almost 3-fold increase in the antioxidant activity). With the ChL or Se treatments, the antioxidant activity was stimulated less efficiently but still significantly, as the application of these elicitors resulted in an approximately 2-fold increase in the ability of the extracts to reduce DPPH, compared to the control.

**Figure 7:**
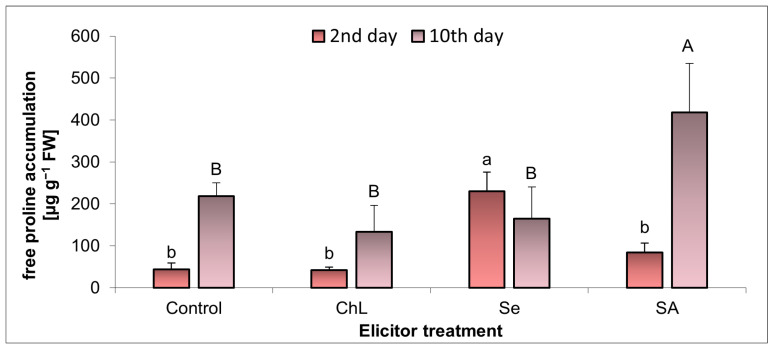
Effect of soil application of ChL—chitosan lactate, Se—sodium selenite, and SA—salicylic acid on free proline accumulation in *C*. *majus* leaves on the 2nd and 10th day of the elicitor exposure. Control plants were treated with distilled water. Values are the means ± standard deviation, shown as positive error bars (n = 4). Different letters (lowercase or capital) indicate statistically significant differences between treatments on the same sampling day (*p* < 0.05, LSD-Fisher test). One-way ANOVA was performed independently for each time point.

The concentration of free proline in the *C. majus* leaves changed depending on the elicitation day and the type of the compound applied (Figure 7). Not all of the elicitors used caused changes in the accumulation of this amino acid. On day 2 after the application of Se, the free proline concentration increased 4-fold, compared to the control plants. In turn, on day 10 after the application of SA, the content of free proline increased by 91%. No significant effect of ChL on the level of this amino acid was observed at the analyzed time points.

### 2.3. Selected Biometric and Physiological Parameters of C. majus Treated with Different Elicitors

The application of the elicitors generally did not have a significant effect on the biomass of the *C. majus* shoots (Figure S3) or on the chlorophyll fluorescence parameters (Figure S4a,b). Initially, on elicitation day 4, there were no statistically significant changes in the contents of photosynthetic pigments in the leaves (Figure S5a). However, on day 10 after the application of the elicitors, the contents of chlorophyll *a* and *b* were reduced by Se, as their levels decreased by 32% and 26%, respectively, in comparison with the control (Figure S5b). In turn, the compounds used had no significant effect on the content of carotenoids (Figure S5a,b).

## 3. Discussion

In our study, the effects of the soil application of three chemically different metabolic modulators (chitosan lactate, selenium, salicylic acid) on selected physiological parameters and secondary metabolism of *C. majus* were analyzed. *C. majus* is an important medicinal plant known for its rich content of bioactive compounds, particularly isoquinoline alkaloids, which are largely responsible for the multi-directional pharmacological effects of this species. Research has extensively investigated its biological activities, focusing on antioxidant, anti-inflammatory, antimicrobial, antiparasitic, insecticidal, and anticancer properties. However, to date, there has been little focus on optimizing the production of this species. The optimization of culture conditions may help to develop standardized cultivation practices to produce high-yielding, high-quality *C. majus* raw materials with the enhanced accumulation of bioactive compounds, providing a sustainable source for pharmaceutical applications [18]. Our research can help fill the gap in this area.

Comprehensive research is underway on how to effectively increase the accumulation of bioactive compounds in medicinal plants, including the use of the elicitation method. Despite certain progress, the literature still lacks a thorough exploration, particularly in vivo studies, of the effects of elicitors representing chemically diverse metabolite classes on primary and secondary metabolism across various plant species [10,19,20,21]. Furthermore, in the case of medicinal plants, most of the existing research has focused on the elicitation of terpenoids or (poly)phenolic compounds and, less frequently, alkaloids [22,23,24,25].

Our in vivo research is the first to comprehensively report the effects of three chemically different metabolic modulators on such a broad profile of alkaloids in *C*. *majus*, six of which were identified in the shoot extracts. Unlike many medicinal plant species where phenolic compounds or terpenoids dominate the phytochemical profile, *C*. *majus* is distinguished by its specific and highly concentrated isoquinoline alkaloid content, particularly in shoots. The predominance of alkaloids not only defines the pharmacognostic identity of the species but also reflects their ecological role in plant defense mechanisms against stress factors [12,15,26]. Their pharmacological and ecological importance justifies a focused approach to enhance their accumulation through elicitation strategies. The study did not show a significant effect of the elicitors used on the accumulation of the dominant alkaloids (coptisine and chelidonine). Nevertheless, we noted that other key alkaloids, such as protopine, berberine, and allocryptopine accumulated more efficiently under the SA and Se treatments. Of the compounds tested, ChL proved to be the least effective in stimulating alkaloid biosynthesis, with only allocryptopine levels increasing 4 days after its application. Although similar research on the elicitation of *C. majus* is still insufficient and even marginal, several strategies have been considered to enhance the accumulation of its alkaloids. For instance, a greenhouse study analyzed the effects of drought stress, methyl jasmonate (MeJa), and SA on the concentration of a specialized metabolite in three plant species, including *C. majus*. It was found that both the moderate drought stress and the MeJa application had a beneficial impact on the total alkaloid content in *C. majus* [19]. Similarly, in vitro studies demonstrated a significant increase in chelidonine and sanguinarine under MeJa treatment [18].

The optimization of strategies for stimulating the production of major alkaloids with medicinal properties requires a deeper understanding of their biosynthetic pathways. Encouragingly, the first experimental studies are emerging that use *C. majus* as a model system in research on the biosynthesis and accumulation of isoquinoline alkaloids. The issue has been addressed by Yahyazadeh et al. [26], who examined the effects of drought and salinity stress on alkaloid accumulation in *C. majus*. The study reported a significant increase in dihydrocoptisine biosynthesis under stress, which was associated with increased transcript levels of the gene encoding stylopine synthase—a crucial enzyme in alkaloid biosynthesis. Expanding on the influence of abiotic elicitors on the contents of other alkaloids, another in vivo experiment demonstrated the effects of the foliar application of SA in *Catharanthus roseus* in salt-stress and non-stress conditions [27]. The results showed that SA reduced the adverse effects of salinity while improving plant growth and stimulating the total concentration of indole alkaloids, vinblastine, and vincristine. In turn, Farouk et al. [28] investigated various elicitor treatments in *Vinca minor* pot cultivation. The study demonstrated that the foliar application of chitosan combined with the endophyte *Streptomyces* sp. enhanced plant growth, the chlorophyll content, the antioxidant capacity, and the accumulation of different phytochemicals (phenols, flavonoids, anthocyanins, and alkaloids). The elicitors tested in our study also effectively increased total levels of phenolic compounds (but not soluble flavonols), even up to 2-fold, compared to the respective controls. Simultaneously, all the elicitor treatments used in the experiment resulted in enhanced antioxidant activity of the *C. majus* shoot extracts, which is a relatively common effect observed with an increase in the phenolic compounds. The greatest increase (approximately 3-fold) in the ability of the extracts to reduce DPPH, compared to the control, was noted on day 10 after the SA treatment, but the ChL or Se application also stimulated the antioxidant activity (approx. 2-fold increase, compared to the control). As mentioned above, studies focusing on alkaloid stimulation have been less common, but other examples from the literature indicate that elicitors are increasingly being used in extensive in vivo studies. In an experiment conducted by Sayed and Ahmed [29], three types of elicitors (gamma irradiation, nano-selenium, chitosan) and fertilizers (NPK, Moringa extract, humic acid) were used in vivo in *Artemisia* plants, and chitosan was proposed as the most effective, compared to the other metabolic modulators tested. Although both chitosan and nano-selenium (in foliar application) were particularly effective in increasing the artemisinin content and the quantity and quality of essential oil in *Artemisia annua*, they also acted synergistically with the fertilizers used. Another experiment demonstrated that the foliar application of SA in *Silybum marianum* not only had a positive effect on plant biometric and physiological parameters, but also significantly stimulated the accumulation of silymarin and silybin (A + B) [30].

In response to stress factors, the accumulation of reactive oxygen species (ROS) and ROS-induced changes in the oxidative status of plants are common. ROS play a crucial role in the plant defense mechanisms, but their overproduction can be toxic, despite the effective protection from a complex antioxidant system [31,32]. There is a relationship between ROS and proline accumulation in plants. Since both are synthesized in response to stress, it is suggested that proline acts as a non-enzymatic antioxidant against ROS and has been identified as a key molecule in mitigating oxidative stress by scavenging hydroxyl radicals to protect cells from oxidative damage. However, its direct role as a ROS scavenger is debated. Some studies indicate that proline does not directly quench certain ROS, e.g., singlet oxygen. Surprisingly, proline metabolism may also contribute to the generation of ROS [32,33,34]. Not all of the metabolic modulators tested in our study increased the accumulation of free proline; it also depended on the time of exposure to the elicitors. Comparing the most noticeable changes, the Se application caused a significant increase in proline accumulation after 4 days, but the strongest effect in the SA treatment was observed after 10 days. However, it should be emphasized that the abiotic elicitors (Se and SA) caused these changes, and not ChL.

ROS-induced changes are assigned with their function as secondary messengers, and they are interrelated with the production of specialized metabolites [35]. In a study conducted by Stasińska-Jakubas et al. [21], the foliar application of ChL, Se, and SA resulted in a significant increase in the accumulation of phenolic metabolites in *Melissa officinalis* shoots, which was accompanied by elevated levels of H_2_O_2_ and O_2_^−^˙, as well as enhanced lipid peroxidation. Similarly, these elicitors tested in *Hypericum perforatum*, especially Se and SA, enhanced the accumulation of phenolic compounds and selected flavonoids. At the same time, an increase in the level of free proline and O_2_^−^˙ and modulation of the activity of selected antioxidant enzymes were found [36]. The above studies were carried out using the foliar application of metabolic modulators, which was not possible with the hydrophobic leaves of *C*. *majus*, and another method of application could have significantly influenced the plant response. In the present study, the relationship between the redox status of plants and the levels of specialized metabolites cannot be clearly highlighted. Not all of the elicitors used in our experiment caused significant changes in the oxidative parameters, with Se and SA appearing to induce plant responses more effectively. The accumulation of H_2_O_2_ in the *C. majus* leaves was strongly enhanced by Se and SA, especially after 10 days of the elicitor treatment. Nevertheless, these changes were not related to lipid peroxidation, since at the same time, the TBARS concentration did not change or even decreased by 40% under the influence of SA. On the other hand, all of the elicitors tested enhanced the accumulation of TBARS to a similar extent shortly after their application. These results are most likely related to the functioning of an efficient antioxidant system in plants, which allows ROS species to function as signaling molecules but prevents their long-term harmful effects. Comparing these results with the activity of the analyzed antioxidant enzymes, the strongest response was observed at 10 days after the soil application of Se, which caused a 15-fold increase in GPOX activity in relation to the control plants.

The results obtained suggest that the observed enhancement of the antioxidant capacity of *C*. *majus* extracts following Se and SA elicitation is at least partially associated with the increased accumulation of TPC. These compounds are known to act as non-enzymatic antioxidants, contributing to the plant’s defense mechanisms under stress [34,35]. At the same time, both elicitors induced changes in the plant’s oxidative status, including elevated levels of H_2_O_2_ and intensified lipid peroxidation, particularly shortly after application. This indicates a transient pro-oxidant effect that may trigger the biosynthesis of secondary metabolites, in line with established models of elicitor-induced ROS signaling [34,36]. The concomitant accumulation of free proline, a well-recognized stress marker and ROS modulator, can further support this interpretation [32,33]. Thus, our findings suggest a correlation between an elicitor-induced oxidative imbalance and the activation of antioxidant pathways and phenolic biosynthesis, which is consistent with observations reported in other medicinal plant species [36].

Despite the visible dependence of the analyzed parameters on the duration of elicitation, it is still difficult to indicate its optimal time. Although the most significant changes were generally observed on the 7th and 10th day, it should be emphasized that the results are too complex and dependent on the type of elicitor. This study also indicates a clear advantage of the abiotic elicitors used over ChL in *C. majus* elicitation. Together, these findings highlight the need for further investigation into the complex interactions between chemical, especially abiotic, elicitors and plant metabolic pathways to optimize the production of alkaloids in medicinal plants, including *C. majus* as a model species for the biosynthesis of isoquinoline alkaloids.

## 4. Materials and Methods

### 4.1. Plant Growth Conditions and Experimental Layout

The experiment was conducted in an air-conditioned phytotron equipped with LED lamps (photosynthetic photon flux density, 170–200 µmol m^−2^ s^−1^; temperature, 26/22 °C day/night; relative humidity, 60–65%) with an initial photoperiod of 14/10 h followed by 16/8 h (day/night). The plants were grown in plastic pots (0.5 L) filled with a universal soil substrate (COMPO BIO, Kronen, Kehl am Rhein, Germany; pH 6.0). Greater celandine (*C*. *majus*) seeds were purchased from a certified local supplier (HORTICO S.A., Wroclaw, Poland) and sown in pots (25 seeds in each). They were sprayed abundantly with distilled water and left to germinate. After 3 weeks, the plants were pricked out so that each pot contained 8 seedlings with equal growth. During seed germination and plant growth, they were regularly watered with an appropriate volume of distilled water. After 5 weeks of cultivation, the photoperiod was extended from 14 to 16 h, and all the plants were uniformly watered with Hoagland’s nutrient solution [37].

The experimental variants were established at 10 weeks after sowing the seeds. Due to the strongly hydrophobic layer of epicuticular waxes on the surfaces of *C*. *majus* leaves, the water solutions of the tested elicitors were applied into the soil in a volume of 30 mL per pot. Based on available literature data and our previous laboratory research results, four experimental combinations were established and treated with the following solutions: control (distilled water), 100 mg/L chitosan lactate (ChL; Heppe Medical Chitosan, Halle, Germany, deacetylation degree: 80–95%), 10 mg/L selenium (Se; Na_2_SeO_3_·5H_2_O; Fluka, Geneva, Switzerland), and 150 mg/L salicylic acid (SA; PoCH, Gliwice, Poland). Consequently, there were four treatments, with four pots per treatment, resulting in 32 plants per treatment group.

All plants were at the same physiological stage (10 weeks after sowing) at the start of the elicitor treatment. Sampling at predefined time points was part of a time-course design aimed at monitoring the dynamics of biochemical and physiological responses. Plant material for the determination of secondary metabolites and free radical-scavenging activity (FRSA) was collected on days 4, 7, and 10 after elicitation treatment, corresponding to the expected timing of secondary metabolite accumulation. In turn, oxidative status parameters (H_2_O_2_ concentration, lipid peroxidation level, activity of antioxidant enzymes) and physiological indicators (photosynthetic pigment content, chlorophyll *a* fluorescence, free proline content) were assessed on days 2 and 10 to capture both early and late responses. The entire dataset was obtained from a single experimental setup conducted under strictly controlled environmental conditions.

### 4.2. Preparation of Methanol Extracts from Shoots

To prevent the loss of bioactive compounds, *C*. *majus* shoots were slowly dried at room temperature in a dark place. The material was then ground in a porcelain mortar into a fine powder.

Metabolites from the plant material were extracted using an Accelerated Solvent Extractor (ASE, Thermo Scientific Dionex, ASE 350, New York, NY, USA). Approximately 750 mg of ground, dry shoots were placed in a 22 mL stainless steel extraction cell. Methanol (100%) was used as the solvent. The extraction was carried out at 80 °C for 15 min under a constant pressure of 1500 psi. The resulting crude extracts were directly analyzed via high-performance liquid chromatography with a diode array detector (HPLC-DAD) and used for TPC and TSF determination.

### 4.3. Determination of Selected Secondary Metabolites in Plant Extracts

#### 4.3.1. Quantitative and Qualitative Determination of Alkaloids

##### Reference Standards and Chemicals

Alkaloid standards: allocryptopine, hydrochloride of chelidonine and protopine, chloride of berberine, chelerythrine, coptisine, and sanguinarine were purchased from Sigma-Aldrich (St. Louis, MO, USA). Ammonium acetate, acetic acid, and HPLC-grade acetonitrile were purchased from Merck (Darmstadt, Germany). Water was deionized and purified using an ULTRAPURE Milipore Direct-Q^®^3UV–R (Merck).

##### HPLC-DAD and UHPLC-ESI-TOF-MS Analysis

Alkaloids were determined according to the previously published methodology [38], as follows: Chromatograph VWR Hitachi Chromaster 600 (Hitachi, Tokyo, Japan) with a spectrophotometric detector (DAD) and EZChrom Elite software ver. 3.3.2 (Merck); XB-C18 reversed-phase core-shell column (Kinetex, Phenomenex, Aschaffenburg, Germany) (25 cm × 4.6 mm i.d., 5 μm particle size), at a temperature of 25 °C; mobile phase, acetonitrile (solvent A) and 10 mM aqueous solution of ammonium acetate adjusted to pH = 4 with acetic acid (solvent B); gradient program, A 20%, B 80% for 0–20 min, A 25%, B 75% for 20–27 min and then A 30%, B 70% for 27–60 min; eluent flow rate 1.2 mL min^−1^. Compounds in plant extracts were identified based on a comparison of retention times and UV-Vis spectra with corresponding standards (Figure S1). Quantitative analysis was performed at λ = 288 nm for protopine, allocryptopine, and chelidonine, λ = 357 nm for coptisine, λ = 327 nm for sanguinarine, λ = 344 nm for berberine, and λ = 268 nm for chelerithrine. Data were recalculated per gram of plant dry weight (DW). The limits of detection (LODs) and quantification (LOQs) for the seven target alkaloids were determined based on signal-to-noise ratios of 3:1 and 10:1, respectively (Table S1).

To further confirm the identity of the detected alkaloids and strengthen dereplication, selected samples were analyzed using ultra-high-performance liquid chromatography coupled with electrospray ionization time-of-flight mass spectrometry (UHPLC-ESI-TOF-MS). The system consisted of an Agilent Infinity II Series UHPLC coupled with an ESI/TOF detector (Agilent Technologies, Santa Clara, CA, USA), using a reversed-phase Kinetex C18 column (150 mm × 2.1 mm, 1.7 μm particle size; Phenomenex, Torrance, CA, USA). The mobile phase was composed of solvent A (water with 0.05% formic acid) and solvent B (acetonitrile with 0.05% formic acid), delivered at a flow rate of 0.2 mL min^−1^. MS analyses were performed in positive ion mode under the following conditions: drying gas temperature of 325 °C, gas flow of 8 L min^−1^, nebulizer pressure of 30 psi, capillary voltage of 3500 V, skimmer voltage of 65 V, and fragmentor voltage of 200 V. The MS data confirmed the identities of compounds detected via HPLC-DAD by comparing molecular ions ([M + H]^+^) with standard *m*/*z* values (Table S2, Figure S2).

#### 4.3.2. Total Phenolic Compounds

The concentration of TPCs in the methanolic extracts prepared previously (2.2) was examined using spectrophotometry (UV-VIS spectrophotometer Cecil CE 9500, Cecil Instruments, Cambridge, UK) at 756 nm. The content of TPC was determined using the Folin–Ciocalteau reagent according to the method proposed by Wang et al. [39] with slight modifications described previously [21]. The content of TPC was calculated from the standard curve of gallic acid and expressed as gallic acid equivalents (GAEs).

#### 4.3.3. Total Soluble Flavonols

The content of TSF in the plant extracts (Section 4.2) was determined as a complex with aluminum ions following the simplified method proposed by Christ-Müller [40] described previously [21]. The absorbance of the solutions was measured at a 425 nm wavelength (UV-VIS spectrophotometer Cecil CE 9500, Cecil Instruments, Cambridge, UK). The concentration of TSF was calculated from the standard curve of rutin and expressed as rutin equivalents (RUE).

### 4.4. Determination of the Oxidative Status

#### 4.4.1. H_2_O_2_ Accumulation

The H_2_O_2_ level in the leaves of the control and elicitor-treated plants was determined using the method described by Jena and Choudhuri [41] on days 2 and 10 of elicitation. The leaf samples from the middle part of the shoot were homogenized in phosphate buffer and centrifuged. Next, 1.5 mL of the supernatant and 0.5 mL of 0.1% TiO_2_ in H_2_SO_4_ were mixed and centrifuged again. The yellow color intensity of the reaction mixture was measured spectrophotometrically at λ = 410 nm (UV-VIS spectrophotometer Cecil CE 9500, Cecil Instruments, Cambridge, UK). Based on the absorbance value and the molar absorbance coefficient (0.28 μM^−1^ cm^−1^), the content of H_2_O_2_ was calculated. The detailed procedure was described previously [21].

#### 4.4.2. Lipid Peroxidation Level

The concentrations of TBARS (thiobarbituric acid reactive substances) indicating the level of membrane lipid peroxidation were determined in leaf samples collected from the middle part of the shoot on days 2 and 10 of elicitation using a modified method developed by Heath and Packer [42]. The samples were ground in a trichloroacetic acid solution and centrifuged. Next, 20% trichloroacetic acid containing 0.5% thiobarbituric acid supplemented with butylated hydroxytoluene was added to the supernatant. The reaction mixture was heated and centrifuged again, and its absorbance was determined spectrophotometrically at 600 and 532 nm (UV-VIS spectrophotometer Cecil CE 9500, Cecil Instruments, Cambridge, UK). The concentration of TBARS was determined based on the molar absorbance coefficient (155 mM^−1^ cm^−1^).

#### 4.4.3. Antioxidant Enzyme Activity

The activity of guaiacol peroxidase (GPOX, EC 1.11.1.7), catalase (CAT, EC 1.11.1.6), and ascorbate peroxidase (APOX, EC 1.11.1.11) in the leaves of *C*. *majus* were conducted on days 2 and 10 of elicitation. Leaf samples were taken from the middle part of the shoot. CAT activity was determined following the method described by Golan et al. [43] at a wavelength of 240 nm. GPOX activity was assayed with the method used by Małolepsza et al. [44], and absorbance was measured at a wavelength of 480 nm for 4 min at 1 min intervals. APOX activity was assayed using the technique proposed by Nakano and Asada [45]; it was measured at 290 nm for 5 min at 1 min intervals. Detailed descriptions of these methods were described previously Stasińska-Jakubas et al. (2024) [36]. The activity of the enzymes was determined spectrophotometrically with the use of a Cecil CE 9500 device.

#### 4.4.4. Free-Radical-Scavenging Activity

The FRSA of the *C*. *majus* methanol leaf extracts (Section 4.2) was performed spectrophotometrically at 517 nm using a 200 μM solution of synthetic radical DPPH (1,1-diphenyl-2-picrylhydrazyl) dissolved in 80% methanol. The analysis and calculations were carried out according to the Molyneux (2004) [46] method described in detail previously [21].

### 4.5. Determination of Plant Growth, Selected Physiological Parameters, and Free Proline Accumulation

#### 4.5.1. Fresh Weight of Shoots

The fresh weight of *C*. *majus* shoots was determined on the final day of the experiment (10 days after the application of metabolic modulators). The shoots were cut off a few millimeters above the surface of the soil substrate and weighed on a laboratory scale. The results are expressed in grams per plant.

#### 4.5.2. Content of Photosynthetic Pigments

The concentration of photosynthetic pigments in the leaves of *C*. *majus* was determined spectrophotometrically on days 2 and 10 after the application of metabolic modulators following the Lichtenthaler and Wellburn [47] method and calculations. For these analyses, leaves located in the central part of the shoot were sampled and homogenized in 80% acetone. The absorbance of the solutions was measured at 663 nm, 645 nm, and 470 nm (UV-VIS spectrophotometer Cecil CE 9500, Cecil Instruments, Cambridge, UK).

#### 4.5.3. Measurement of Selected Parameters of Chlorophyll *a* Fluorescence

Selected chlorophyll *a* fluorescence parameters were measured on leaves located in the central part of the shoot on days 2 and 10 after the treatment with elicitors. The following parameters were measured: F0—minimum fluorescence, Fm—maximum fluorescence, and Fv/Fm—ratio of variable fluorescence to maximum fluorescence using a Handy-PEA portable photofluorimeter (Hansatech Instruments, Norfolk, UK) as described previously [21].

#### 4.5.4. Free Proline Accumulation

The accumulation of proline in the *C*. *majus* leaves was determined on elicitation days 2 and 10 with the method described by Bates et al. [48]. For these analyses, leaves located in the central part of the shoot were sampled. The absorbance was measured spectrophotometrically at 520 nm (UV-VIS spectrophotometer Cecil CE 9500, Cecil Instruments, Cambridge, UK). The proline concentration was read from a standard curve prepared for pure proline standard and converted to the fresh weight.

### 4.6. Statistical Analysis

The experiment was established using a completely randomized design. All numerical data provided by the laboratory analyses were subjected to one–way analysis of variance (ANOVA) to compare the effects of elicitor treatments within each sampling day. The LSD-Fisher test was applied for post hoc comparisons at a significance level of *p* < 0.05. Values expressed as a percentage (reduction of the DPPH) were normalized through arcsin transformation prior to ANOVA.

A time-point-specific approach was chosen to enable a more detailed evaluation of elicitor effects at each stage of the experiment, rather than assessing treatment × time interactions. Although a two-way ANOVA was initially considered, preliminary analyses revealed that temporal physiological variation could introduce confounding effects, potentially obscuring biologically meaningful treatment-specific responses. Therefore, one-way ANOVA was deemed the most appropriate method, better reflecting both the structure and objectives of the study.

## 5. Conclusions

The present study reveals, for the first time, the distinct biochemical and physiological responses of *C*. *majus* to different chemically diverse elicitors applied in vivo via the soil substrate. An integrated experimental design was employed, including secondary metabolite profiling, oxidative stress biomarkers, and physiological indicators, all assessed under uniform environmental conditions and including the exposure time. Given the hydrophobic nature of *C*. *majus* leaves, the application of chemical elicitors to the soil substrate appears to be the only possible technique for use in vivo. Among the tested compounds, Se and SA (abiotic elicitors), led to the significant modification of the concentration of valuable specialized metabolites in the shoots of *C*. *majus*. Although chelidonine and coptisine, the dominant alkaloids in this species, were not substantially affected (except for SA, which increased chelidonine levels on day 10 after application), enhancing other bioactive constituents demonstrates the elicitor’s selective action and its practical implications. From a pharmacological perspective, the stimulated compounds are known for their cytotoxic, antimicrobial, and hepatoprotective activities, which may enhance the therapeutic potential of *C*. *majus* raw materials. The elicitor treatments also increased the total phenolic content and markedly improved the antioxidant activity of the plant extracts. Although elicitor-induced changes in oxidative status (increased H_2_O_2_ accumulation and enhanced lipid peroxidation) and free proline accumulation were observed, it remains unclear whether these shifts are causally related to the up-regulation of specialized metabolite biosynthesis.

Overall, the results support the potential of soil-applied Se and SA as a promising method for improving the phytochemical value of *C*. *majus* in controlled pot cultivation. However, the complexity of the factors that determine the success of elicitation and the limited information on alkaloid stimulation in this plant species require further research to optimize elicitation in *C*. *majus*, including an analysis of the activity and gene expression of key enzymes involved in isoquinoline alkaloid biosynthetic pathways.

## Data Availability

The datasets generated during the study are available from the corresponding author on the request.

## Supplementary Materials

The following supporting information can be downloaded at: https://www.mdpi.com/article/10.3390/molecules30132782/s1. Figure S1: Sample HPLC chromatogram (A), spectrochromatogram (B) and DAD spectra of the chromatographic peaks (C) obtained during the separation of alkaloids from *C*. *majus* methanolic extracts–protopine (1), allocryptopine (2), chelidonine (3), coptisine (4), berberine (5), and chelerythrine (6); Figure S2: Mass spectra extracted from the chromatographic peaks of reference standards (red traces) and corresponding peaks detected in the *C*. *majus* shoot extract (green traces); Figure S3: Effect of soil application of ChL–chitosan lactate, Se–sodium selenite, and SA–salicylic acid on the biomass of *C*. *majus* shoots after 10 day of the elicitor exposure. Control plants were treated with distilled water. Means (± SD; n = 10) marked with different letters differ statistically significantly (*p* < 0.05, LSD-Fischer test); Figure S4: Effect of soil application of ChL–chitosan lactate, Se–sodium selenite, and SA—salicylic acid on selected parameters of chlorophyll *a* fluorescence in *C*. *majus* on the 2nd (a) and 10th (b) day of the elicitor exposure. Control plants were treated with distilled water. Values are means ± standard deviation, shown as positive error bars (n = 4). Different letters (lowercase, italic, or capital) indicate statistically significant differences between treatments for individual parameters (*p* < 0.05, LSD-Fisher test); Figure S5: Effect of soil application of ChL—chitosan lactate, Se—selenite, and SA—salicylic acid on the concentration of photosynthetic pigments in *C*. *majus* on the 2nd (a) and 10th (b) day of the elicitor exposure. Control plants were treated with distilled water. Values are means ± standard deviation, shown as positive error bars (n = 4). Different letters (lowercase, italic, or capital) indicate statistically significant differences between treatments for individual pigments (*p* < 0.05, LSD-Fisher test). Table S1: Limits of detection and quantification for selected isoquinoline alkaloids determined by HPLC-DAD; Table S2: Comparison of MS data for standards and components of the *C*. *majus* methanolic extract.
