# Supplementary material for: Effect of Soil-Applied Metabolic Modulators on the Accumulation of Specialized Metabolites in Chelidonium majus L
Source: Molecules. 2025 Jun 27;30(13):2782. doi: 10.3390/molecules30132782 (PMC12251457; doi:10.3390/molecules30132782)
Supplement: Supplementary file 1 [file molecules-30-02782-s001.zip › molecules-3641915-supplementary.pdf]

SUPPLEMENTARY MATERIALS

Effect of Soil-Applied Metabolic Modulators on the Accumulation of Specialized Metabolites in *Chelidonium majus* L.

Maria Stasińska-Jakubas [1], Sławomir Dresler [2,3], Maciej Strzemski [2], Magdalena Wójciak [2], Katarzyna Rubinowska [1] and Barbara Hawrylak-Nowak [1,*]

[1] Department of Botany and Plant Physiology, Faculty of Environmental Biology, University of Life Sciences in Lublin, Akademicka 15, 20-950 Lublin, Poland; maria.jakubas@up.lublin.pl (M.S.-J.); katarzyna.rubinowska@up.lublin.pl (K.R.)
[2] Department of Analytical Chemistry, Medical University of Lublin, Chodźki 4a, 20-093 Lublin, Poland; slawomir.dresler@umlub.pl (S.D.); maciej.strzemski@umlub.pl (M.S.); magdalena.wojciak@umlub.pl (M.W.)
[3] Department of Plant Physiology and Biophysics, Institute of Biological Science, Maria Curie-Skłodowska University, 20-033 Lublin, Poland
* Correspondence: barbara.nowak@up.lublin.pl

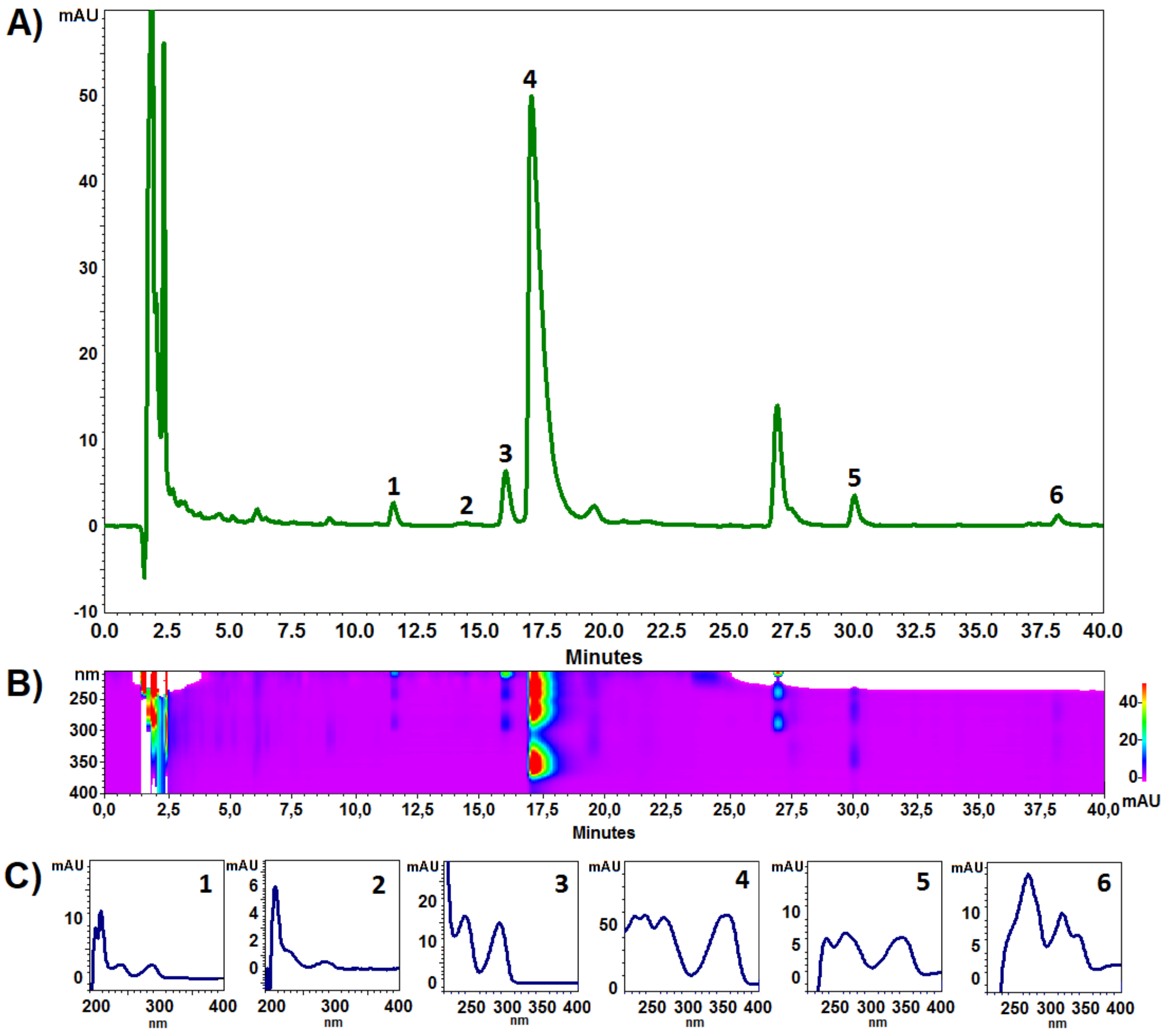

Figure S1. Sample HPLC chromatogram (A), spectrochromatogram (B) and DAD spectra of the chromatographic peaks (C) obtained during the separation of alkaloids from *C. majus* methanolic

SUPPLEMENTARY MATERIALS

extracts – protopine (1), allocryptopine (2), chelidonine (3), coptisine (4), berberine (5), and chelerythrine (6).

Table S1. Limits of detection and quantification for selected isoquinoline alkaloids determined by HPLC-DAD.

Compound	LOD (μg/L)	LOQ (μg/L)
allocryptopine	3.60	11.99
berberine	3.08	10.26
chelerythrine	3.44	11.46
chelidonine	1.26	4.20
coptisine	1.23	4.10
protopine	3.40	11.32
sanguinarine	1.80	5.99

Abbreviations: LOD – limit of detection; LOQ – limit of quantification; HPLC-DAD – high-performance liquid chromatography with diode array detection.

Table S2. Comparison of MS data for standards and components of the *C. majus* methanolic extract.

	R_T (min.)	$(m/z+H)^+$	Δ ppm	formula	Identified
Standard	18.86	354.13450	2.55	$C_{20}H_{19}NO_5$	protopine
Component of the extract	18.98	354.13304	-1.58		
Standard	25.29	370.16587	2.63	$C_{21}H_{23}NO_5$	allocryptopine
Component of the extract	25.18	370.16498	0.22		
Standard	25.85	354.13220	-3.96	$C_{20}H_{19}NO_5$	chelidonine
Component of the extract	25.68	354.13396	1.02		
Standard	27.92	320.09221	1.49	$C_{19}H_{13}NO_4$	coptisine
Component of the extract	27.99	320.09251	2.43		
Standard	31.19	332.09230	1.71	$C_{20}H_{13}NO_4$	sanguinarine*
Component of the extract	31.26	332.09095	-2.37		
Standard	31.87	336.12392	2.64	$C_{20}H_{17}NO_4$	berberine
Component of the extract	31.92	336.12310	0.20		
Standard	33.98	348.12336	0.94	$C_{21}H_{17}NO_4$	chelerythrine
Component of the extract	34.01	348.12214	-2.58		

*sanguinarine – detected but not quantified (below limit of quantification)

SUPPLEMENTARY MATERIALS

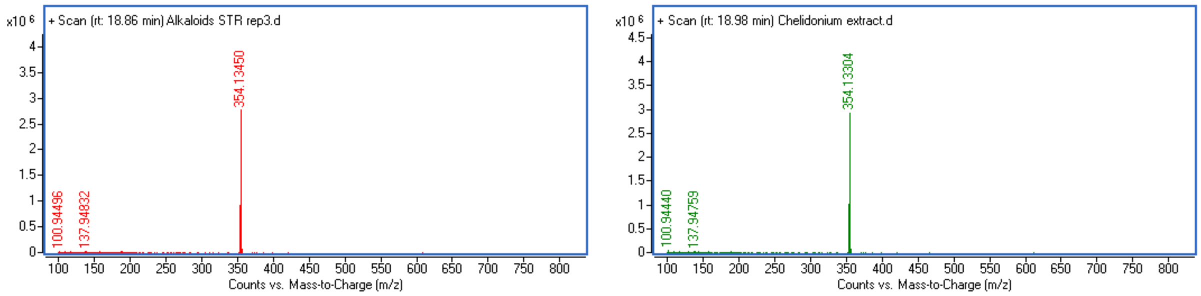

estimated formula: $C_{20}H_{19}NO_5$ - protopine

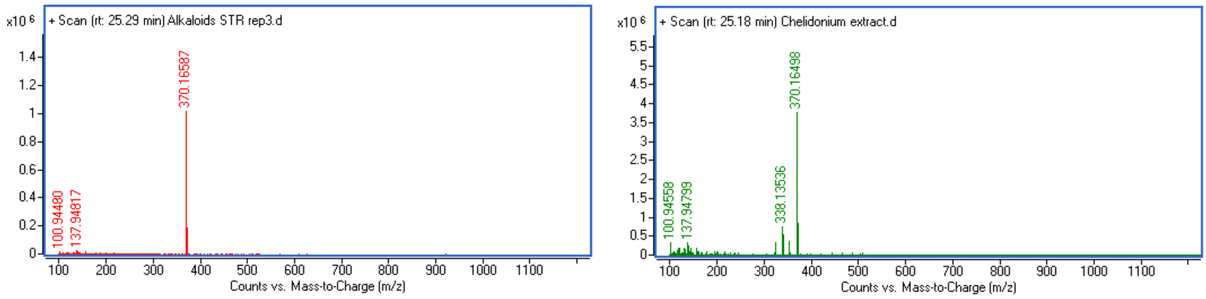

estimated formula: $C_{21}H_{23}NO_5$ – allocryptopine

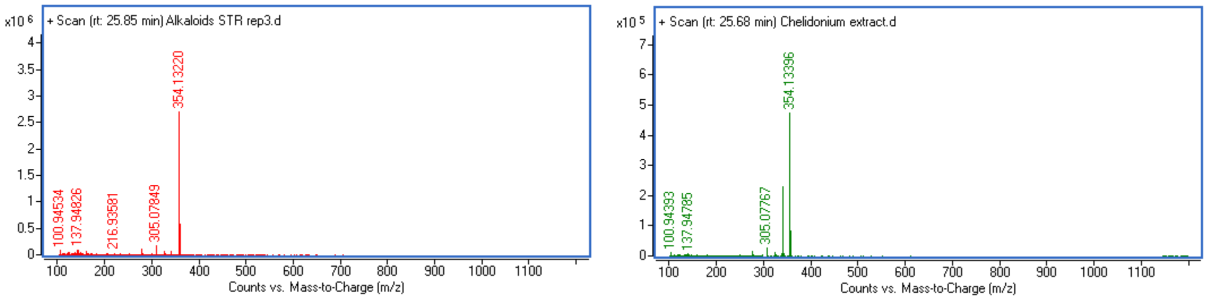

estimated formula: $C_{20}H_{19}NO_5$ – chelidonine

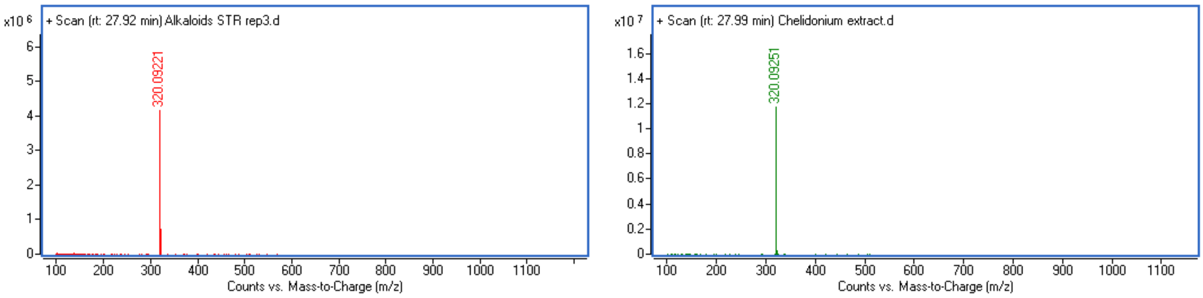

estimated formula: $C_{19}H_{13}NO_4$ – coptisine

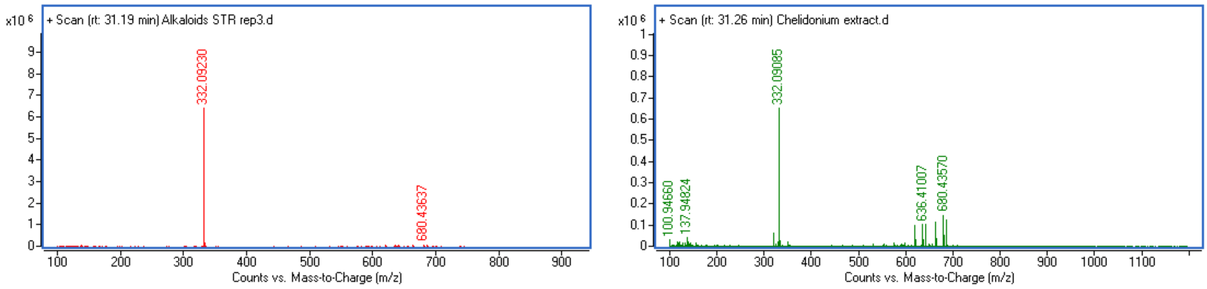

estimated formula: $C_{20}H_{13}NO_4$ - sanguinarine

SUPPLEMENTARY MATERIALS

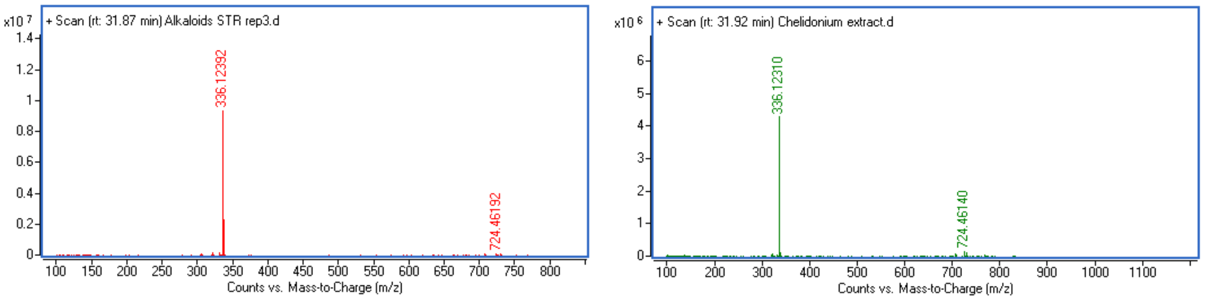

estimated formula: $C_{20}H_{17}NO_4$ – berberine

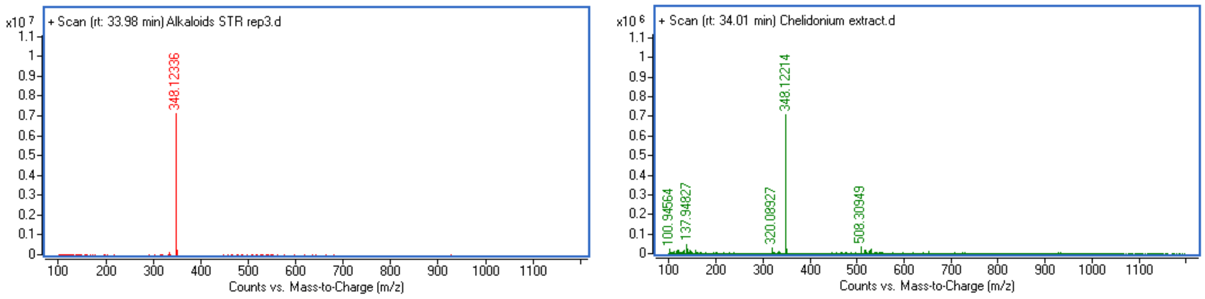

estimated formula: $C_{21}H_{17}NO_4$ – chelerythrine

Figure S2. Mass spectra extracted from the chromatographic peaks of reference standards (red traces) and corresponding peaks detected in the *C. majus* shoot extract (green traces).

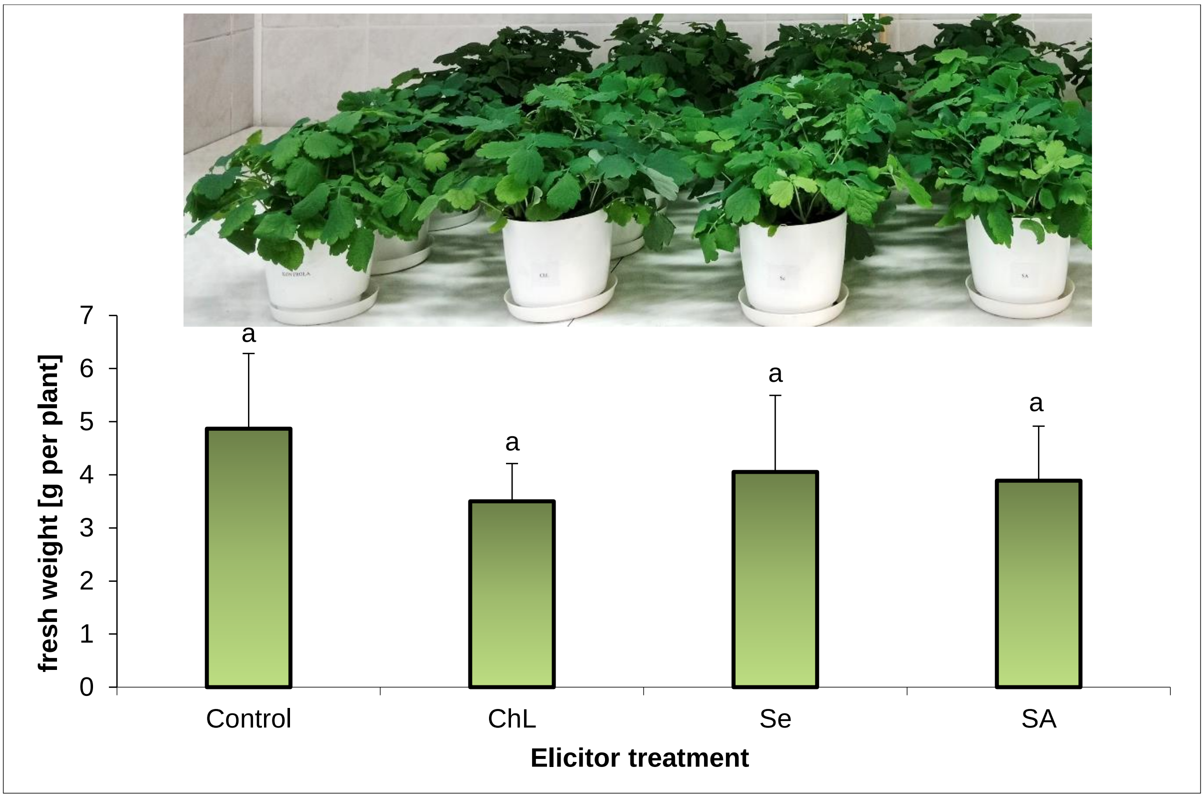

Figure S3. Effect of soil application of ChL – chitosan lactate, Se – sodium selenite, and SA – salicylic acid on the biomass of *C. majus* shoots after 10 day of the elicitor exposure. Control plants were treated with distilled water. Means (± SD; n = 10) marked with different letters differ statistically significantly ($p < 0.05$, LSD-Fischer test).

SUPPLEMENTARY MATERIALS

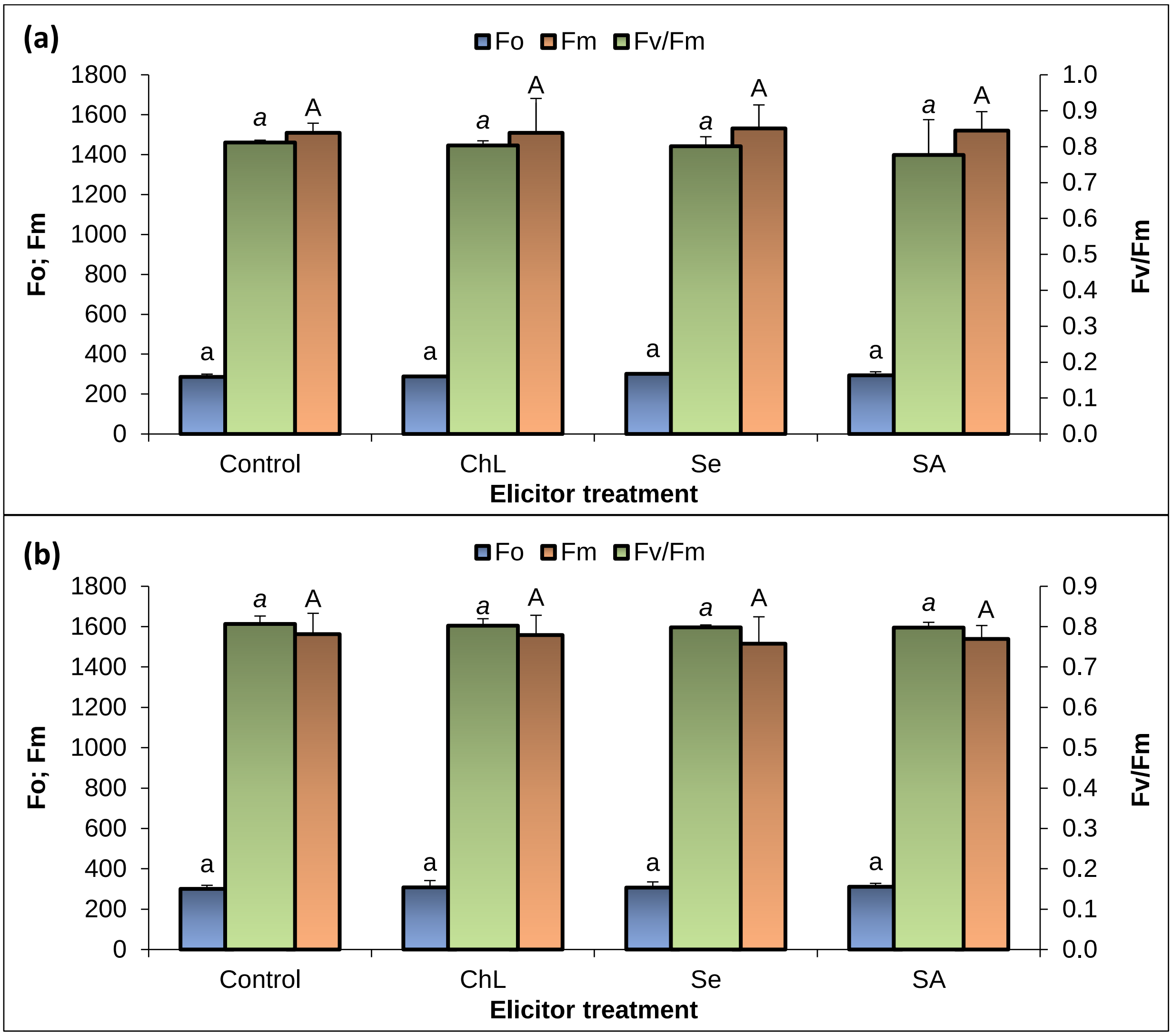

Figure S4. Effect of soil application of ChL – chitosan lactate, Se – sodium selenite, and SA – salicylic acid on selected parameters of chlorophyll *a* fluorescence in *C. majus* on the 2nd (a) and 10th (b) day of the elicitor exposure. Control plants were treated with distilled water. Values are means ± standard deviation, shown as positive error bars (n = 4). Different letters (lowercase, italic, or capital) indicate statistically significant differences between treatments for individual parameters ($p < 0.05$, LSD-Fisher test).

SUPPLEMENTARY MATERIALS

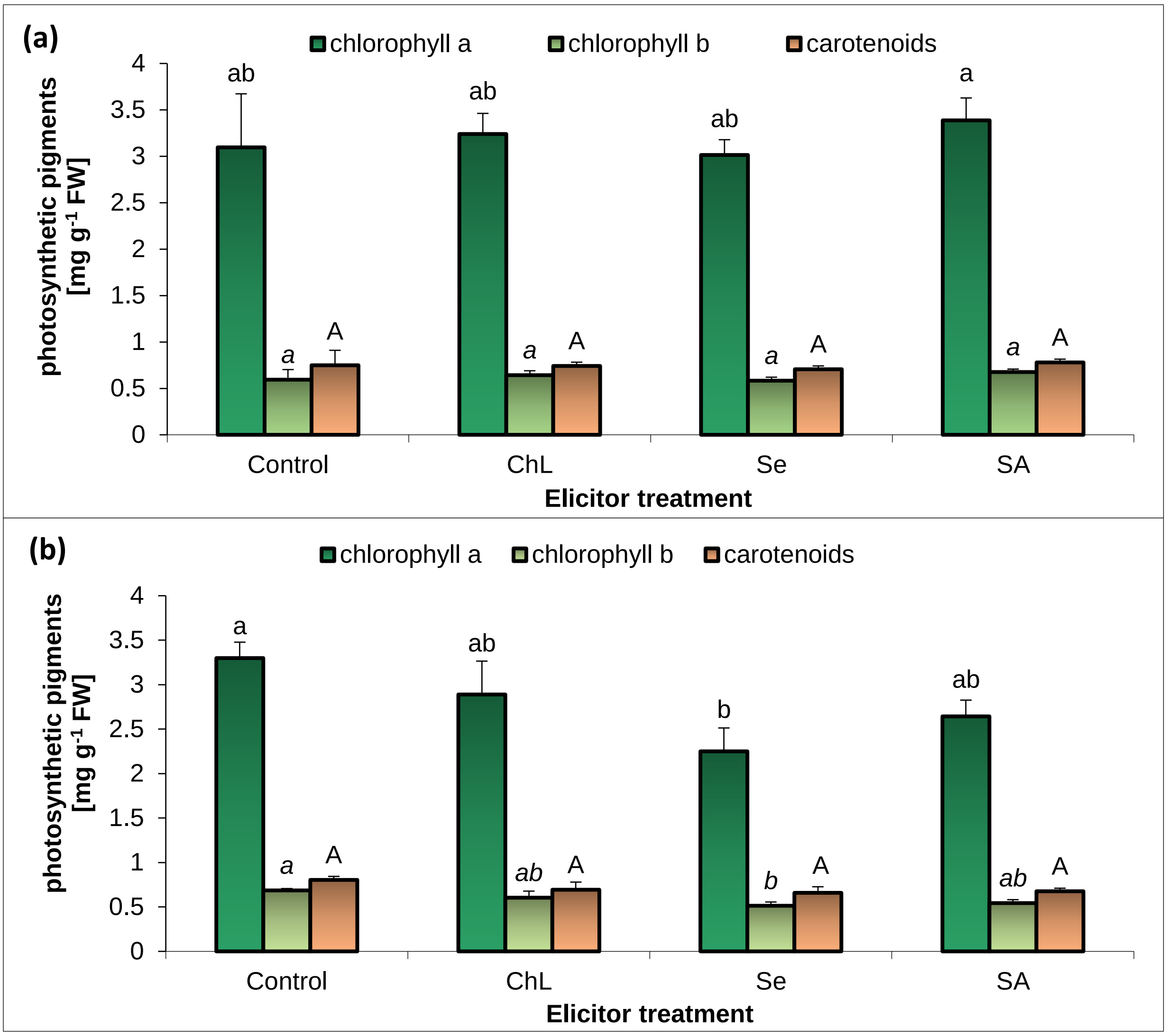

Figure S5. Effect of soil application of ChL – chitosan lactate, Se – selenite, and SA – salicylic acid on the concentration of photosynthetic pigments in *C. majus* on the 2nd (a) and 10th (b) day of the elicitor exposure. Control plants were treated with distilled water. Values are means ± standard deviation, shown as positive error bars (n = 4). Different letters (lowercase, italic, or capital) indicate statistically significant differences between treatments for individual pigments ($p < 0.05$, LSD-Fisher test).
